# Structural and Theoretical Investigation of Anhydrous 3,4,5-Triacetoxybenzoic Acid

**DOI:** 10.1371/journal.pone.0158029

**Published:** 2016-06-29

**Authors:** Paulo S. Carvalho, Leonardo R. Almeida, João H. Araújo Neto, Ana Carolina Q. D. Medina, Antonio C. S. Menezes, José E. F. Sousa, Solemar S. Oliveira, Ademir J. Camargo, Hamilton B. Napolitano

**Affiliations:** 1 Instituto de Física de São Carlos, Universidade de São Paulo, 13560–970, São Carlos, SP, Brazil; 2 Ciências Exatas e Tecnológicas, Universidade Estadual de Goiás, 75132–400, Anápolis, GO, Brazil; 3 Departamento de Química, Universidade Federal de São Carlos, 13565–905, São Carlos, SP, Brazil; University of Calgary, CANADA

## Abstract

A comprehensive investigation of anhydrous form of 3,4,5-Triacetoxybenzoic acid (TABA) is reported. Single crystal X-ray diffraction, Thermal analysis, Fourier Transform Infrared spectroscopy (FTIR) and DFT calculations were applied for TABA characterization. This anhydrous phase crystallizes in the triclinic $P\bar{1}$ space group (Z' = 1) and its packing shows a supramolecular motif in a classical $R_{2}^{2}(4)$ ring formed by acid-acid groups association. The phase stability is accounted in terms of supramolecular architecture and its thermal behaviour. Conformation search at B3LYP/6-311++G(2d,p) level of theory shows the existence of three stable conformers and the most stable conformation was found experimentally. The reactivity of TABA was investigated using the molecular orbital theory and molecular electrostatic potential. The calculation results were used to simulate the infrared spectrum. There is a good agreement between calculated and experimental IR spectrum, which allowed the assignment of the normal vibrational modes

## Introduction

Solid-state characterization of biologically active compounds plays an important role in all areas of pharmaceutical and material sciences [1–3]. As crystalline materials, the supramolecular chemistry of new solid forms of a molecule have been proving significant contributions to obtaining a more effective and active compounds [4–7]. The architecture of molecules in crystal, even in different solid forms arrangement (*i*.*d*. anhydrous, hydrates, cocrystals, polymorphs), opens the door to design functional materials and also to understanding the recognition process of molecules for biological targets. Furthermore, the characterization of different solid forms of compound has implications for fundamental understanding of the assembly of molecules in the solid state and also in the in the understanding of its solid stability and phase transitions, such as dehydration and conformational changes.

**Fig 1:**
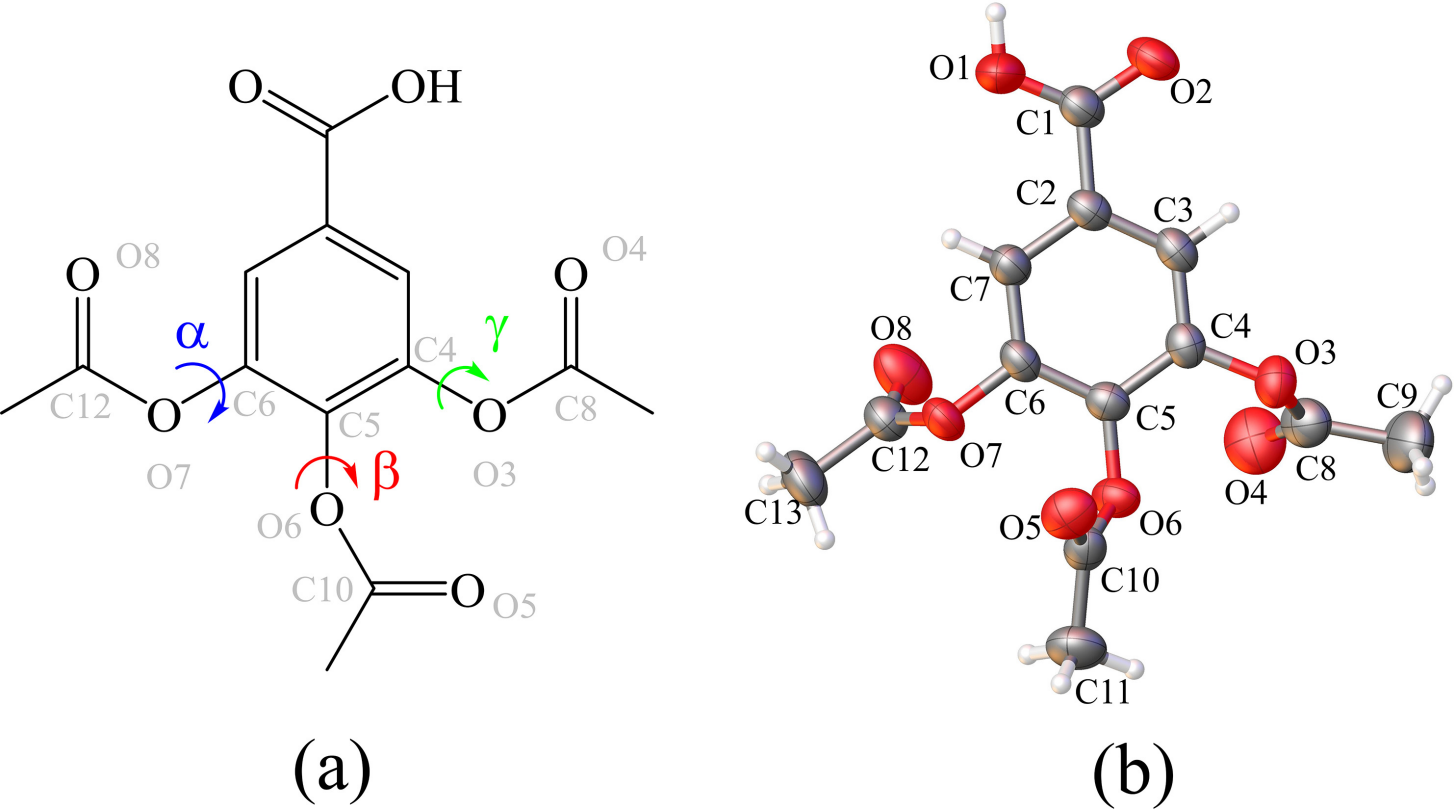
**(a) Structural formula of TABA. Bold bond emphasizes the α (C4−O3−C8−O4), β (C5−O5−C10−O6) and γ (C6−O7−C12−O8) torsion angles. (b) The ORTEP diagram of ellipsoids at 30% probability level with the atomic numbering scheme for asymmetric unit of anhydrous form of TABA**. All bonds are in the normal range and hydrogen atoms are shown as spheres of arbitrary radii. The atomic numbering was used in the theoretical calculation.

In the cocrysallization field, the Gallic acid (GA) and their derivatives represent important cocrystal coformers [8–13] due their ability to form multiple hydrogen bonds (HB) with active pharmaceutical ingredients. Additionally, this class of compound exhibit many significant biological properties, such as antioxidant[14], anti-inflammatory [15], antifungal [16] and carcinogenic [17]. Particularly, esters of GA are attractive chemicals used in the food and pharmaceutical industry, *e*.*g*., in the synthesis of propyl gallate and trimethoprim [18, 19]. Among them, the 3,4,5-triacetoxibenzoic acid, TABA (Fig 1), is a stable molecular form of the GA since all hydroxyl groups are protected as acetates, hence, are less reactive [20, 21]. Noteworthy, this structural modification of GA gives to compound an increase in the effectiveness of the antifungal activity [22]. The TABA exist as a crystalline hydrate [23] in which two conformers are observed in the asymmetric unit. Although to acid-acid supramolecular synthon have recurrent formation between carboxylic acid groups (COOH), in this forms, the water molecule is included in the lattice by the formation of large supramolecular unit with COOH groups. Until now, no anhydrous forms have been reported to TABA.

Considering the TABA functionalities and their potential co-crystallization applications, we report the synthesis, crystallization and solid-state investigation of the anhydrous forms of TABA. In this investigation we use a combination of Single Crystal X-ray Diffraction, Infrared spectroscopy, Differential Scanning Calorimetry (DSC), Thermogravimetric (TGA), Hot Stage Microscopy techniques and DFT calculations to solid-state characterization of compound

## Experimental and Computational Procedure

### Synthesis and crystallization

A mixture of gallic acid (1.88g – 11 mmol), acetic anhydride (10 mL) and H_2_SO_4_ (1 mL, 1 mol.L^-1^) was stirred under reflux for 4 hours. After the cooling, 50 mL of cold water was added to system providing the precipitation of a white crystalline solid. This powder was filtered and washed with water. Then, the resultant solid was further purified by crystallization in methanol at room temperature (25°C). The solvents used in the manipulations were purified by standard methods. The gallic acid were purchased from Sigma-Aldrich®, acetic anhydride and sulphuric acid were purchased from Synth®. Yield: 2.95 g (90%). ^1^H NMR (400.132 MHz, DMSO-*d*_*6*_, 298 K): (ppm): 7.880 (2H, s, *ortho*), 2.327 (3H, s, *para* acetyl) and 2.320 (6H, s, *meta* acetyl). ^13^C NMR (100.62 MHz, CDCl_3_-*d*) δ(ppm): 169.58 (1C, *meta* C = O_acetyl_), 167.68 (2C, *para* C = O_acetyl_), 166.45 (1C, COOH), 143.52, 139.35, 127.46, 122.83 (6C, aromatic), 20.55 (CH_3_ *meta* acetyl), 20.16 (CH_3_ *para* acetyl).

### Crystal structure determination

Single-crystal X-ray diffraction data (SCXRD) for the anhydrous TABA were performed on an Enraf-Nonius Kappa-CCD diffractometer (95 mm CCD camera on κ-goniostat) using graphite-monochromated MoKα radiation (0.71073 Å) at room temperature (298K). Using the Olex2[24] software, the structures were solved by direct methods and the models obtained were refined by full–matrix least squares on *F*^2^ (SHELXTL–97[25]). All the hydrogen atoms were placed in calculated positions and refined with fixed individual displacement parameters [U_iso_(H) = 1.2*U*_eq_ or 1.5*U*_eq_] according to the riding model (C–H bond lengths of 0.97 Ǻ and 0.96 Ǻ, for aromatic and methyl groups, respectively). Molecular representations, tables and pictures were generated by Olex2 [24], MERCURY 3.2 [26] and Crystal Explorer v2.1 [27] programs. The crystallographic information files of the anhydrous TABA molecule (S1, S2, S3, S4, S5, S6, S7, S8 and S9 Tables) were deposited in the Cambridge Structural Data Base [28] under the code CCDC 1470502. Copies of the data can be obtained, free of charge, via www.ccdc.cam.ac.uk.

### Hirshfeld surface analysis

The Hirshfeld surface and the 2D fingerprint plots for the TABA in the anhydrous phase were carried out using Crystal Explorer 3.1[27]. The surface were generated on basis of the normalized contact distances defined: *d*_*i*_ (*i*.*e*., distance from the surface to the nearest atom in another molecule) and *d*_*e*_(*i*.*e*., the distance from the surface to the nearest atom in the molecule itself). The high resolution default of d_*norm*_ surfaces were mapped over the colour scale ranging from −0.71 (red) to 1.37 Å (blue), with the fingerprint plots using the standard 0.6–2.8 Å view of *d*_*e*_ *vs*. *d*_*i*_.

### Thermal Analysis

Thermogravimetric analyses were carried out on a Shimadzu TGA-60 thermobalance. Approximately 3.5 mg of sample were placed on an alumina pan and heated from room temperature up to 200°C, at 10°C·min^-1^, under nitrogen flow (50 mL min^-1^). Differential Scanning Calorimetric measurements were performed on a Shimadzu DSC-60 instrument. Samples (3.5 ± 0.5 mg) were aluminum pans and heated from room temperature up to 400°C, at 10°C·min^-1^, under nitrogen flow (50 mL min^-1^). All data were processed using the Shimadzu TA-60 thermal data analysis software. Microscopy was performed on a Leica DM2500P microscope connected to the Linkam T95-PE hot-stage equipment. Data were visualized with the Linksys 32 software for hot stage control. The crystals of anhydrous TABA acid were placed on a 13 mm glass coverslip, placed on a 22 mm diameter pure silver heating block inside of the stage. The sample was heated at a ramp rate of 10°C/min up to a final temperature of 180°C but discontinued on melting of all material.

### Vibrational spectroscopy

The vibrational spectroscopy in the infrared region was recorded on a FT-IR Bomem Michelson 102 spectrometer in the 4000–250 cm^-1^ region using KBr pellets.

### Computational procedures

As first step, a conformational search was carried out on TABA in order to explore the degrees of freedom of the dihedral angles C4−O3−C8−O4, C5−O5−C10−O6, and C6−O7−C12−O8 using the molecular mechanics method with MM+ force field as implemented in hyperchem 7.5 package of programs. These dihedral angles were varied randomly and simultaneous. The acceptance energy criterion was set up at maximum of 6 kcal/mol above the best. The pre-optimization skipped if the atoms were closer than 0.5 Å and if torsion were within 15^0^ of previous. The structures were considered duplicated if energies were within 0.5 kcal/mol. The search was stopped after 500 interactions. All conformation found in the search and after Gaussian optimization at B3LYP/6-311++G(2d,p) level fell into one of three conformations; namely, TABA-α, TABA-β, and TABA-γ. The further electronic structure computations on TABA were carried out using Gaussian 09 package of programs[29]. Density Functional Theory (DFT) with the 6–311++G(2d,p) basis set Becke’s three parameter hybrid functional(B3)[30] combined with gradient corrected correlation functional Lee-Yang-Parr(LYP) [31] were employed for structural and electronic computations. The dimer geometrical parameters and the dimer interaction energy was carried out at B97D/6-311++g(2d,p) level of theory. The dimer interaction energy with BSSE corrections were computed using the counterpoise method as implemented in g09 program according to formula

$${\Delta E}^{cc}(G)=E_{AB}(G,AB)-[E_{A}(G,AB)+E_{B}(G,AB)],$$ (1)

where Δ*E*^*cc*^(*G*) stands for counterpoise corrected interaction energy and *E*_*A*_(*G*,*AB*) and *E*_*B*_(*G*,*AB*) stand for total energies of monomers *A* and *B* computed with the dimer basis set AB at geometry *G*.

The characterization that the conformers find in a minimal on the hypersurface of the potential energy were accomplished by the absence of imaginary frequencies on the calculated vibrational spectra for the optimized structures. The TABA infrared spectra was carried out using the same level of theory as used for geometry optimizations. The computed electrostatic potential V(**r**) on molecular surface, MEP, shows how the electrons are distributed in three dimensions around a molecule. The V(**r**) created at a point **r** by the nuclei and electrons of a molecule is given by [32]

$$V(r)=\sum_{\alpha }\frac{Z_{\alpha }}{\left|R_{\alpha }-r\right|}-\int \frac{\rho (r^{\prime })}{\left|r^{\prime }-r\right|}dr^{\prime },$$ (2)

where Z_α_ stands for charge on nucleus α located at point **R**_α_ and ρ(**r**′) is the charge density distribution function. V(**r**) is an observable of the wave function and it can be calculated theoretically and measured experimentally by diffraction methods [33]. The MEP surface can be a positive or negative quantity. It will be positive if the positive charges (from nuclei) predominate over the negative charge (from electrons) and conversely. The graphical visualization of the frontier molecular orbitals and the molecular electrostatic potential surface (MEPS) were obtained with the help of Gaussview 5 [34].

## Results and Discussion

### Crystallization and solid state characterization

A notorious characteristic of GA and their derivatives is the tendency to crystallize as hydrates [8,10,11,35–37]. The 3,4,5-triacetoxybenzoic acid (TABA) molecules is a GA derivative that exhibits multifunctional groups represented by carboxylic acid, phenol and ketone (Fig 1) which makes the molecule a strong hydrogen bond acceptor. Depending on the crystallization conditions, it is possible the existence of an anhydrous or a hydrate form of TABA[23]. In our experiments, the crystallization of the TABA from methanol, ethanol and acetone solvents resulted in an anhydrous solid form. A hydrate phase is only obtained when the water is predominant in the crystallization medium, solvent/water ration of 1:2. The anhydrous TABA acid crystallizes in the Triclinic centrosymmetric $P\bar{1}$ space groups with Z' = 1 (Fig 1, Table 1) and has the unit cell volume two time lower than its parent hydrate form [23].

**Table 1:** Crystal data and structure refinement for TABA.

Empirical formula	C_13_H_12_O_8_
Formula weight	296.23
Crystal system	Triclinic
Space group	$P\bar{1}$
a/Å	8.3990(11)
b/Å	8.4870(8)
c/Å	9.8050(11)
α/°	87.999(7)
β/°	82.508(6)
γ/°	88.714(7)
Volume/Å^3^	692.42(14)
Z	2
ρ_calc_/g.cm^-3^	1.421
μ/mm^-1^	0.120
F(000)	308.0
Radiation	MoKα (λ = 0.71073)
2θ range for data collection/°	6.274 to 53.48
Reflections collected	2915
Independent reflections	2915 [R_int_ = 0.038]
Data/restraints/parameters	2915/0/193
Goodness-of-fit on F^2^	1.028
Final R indexes [I > 2σ (I)]	R_1_ = 0.0479, wR_2_ = 0.1313
Final R indexes [all data]	R_1_ = 0.0621, wR_2_ = 0.1417
Largest diff. peak/hole / e Å^-3^	0.30/-0.23

**Fig 2:**
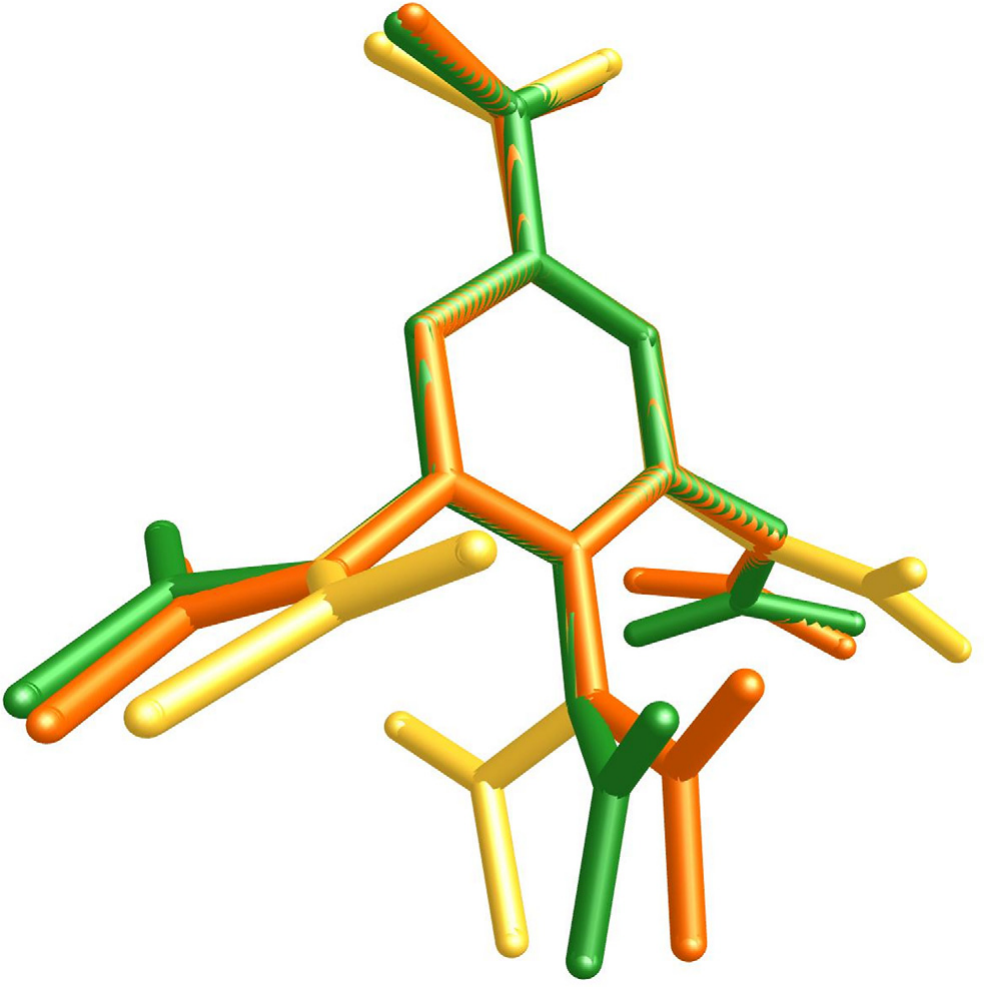
Overlap diagram of TABA from anhydrous (green) and hydrated [23] (yellow shade) forms conformers at their solid-state geometries. The hydrogen atoms were hidden for clarity. The main conformational discrepancies are related with the orientation of acetoxyl groups.

The substituents of aromatic ring of TABA are conformationally flexible. The three acetoxyl groups adopt different synperiplanar orientations with respect to the benzene ring (the α, β and γ torsion angles, Fig 1A, S1 Table) so that there is an alternation in the spatial orientation of groups ketones minimizing the steric effect between them. It is worth noting that TABA exhibit different conformations. The Fig 2 shows the main differences between the TABA acid from hydrated and anhydrous form once superimposing by benzoic moieties. Two conformation are found to TABA and the most relevant differences between them are related to the orientations of the acetoxyl groups. The conformation from the anhydrous form shows agreement with conformers from hydrate one. Since conformational differences are noted to TABA in anhydrous and hydrate forms, the existence of them is related to conformational flexibility of TABA molecule together with consequent variations in the hydrogen bonding schemes of the conformers that provide the inclusion of water molecule into the crystal lattice.

**Fig 3:**
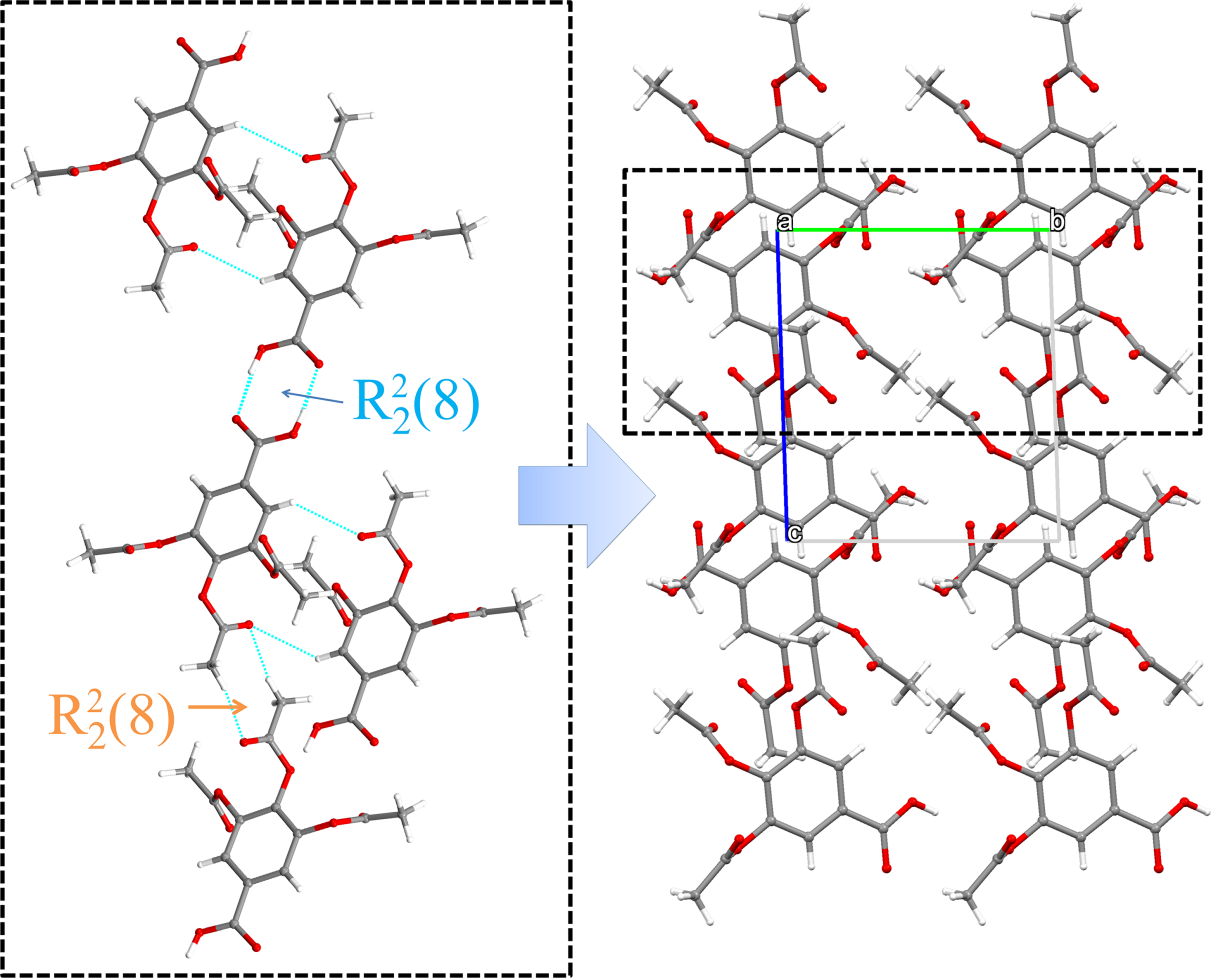
Crystal packing of anhydrous forms of TABA. The main motifs of structure generating different dimeric arrangements.

The molecular packing of anhydrous TABA is stabilized by the presence of dimeric synthons generated by classical and non-classical HBs. The prominent one consists in the usual acid−acid homodimers of graph set $R_{2}^{2}(8)$ forming a 2(TABA) unit. Along the $\left[01\bar{1}\right]$ direction, adjacent units merge each other by of C11−H11B···O6 interaction to form a non-classical $R_{2}^{2}(8)$ motif as shown in Fig 3. Similarly, a dimeric arrangement is formed by the C7−H7···O6 interactions and also by C9−H9A···O8 interactions forming different dimers. The packing view for anhydrous TABA is shown in Fig 3 and Hydrogen bonds are listed in the Table 2.

**Table 2:** Hydrogen bond geometry for TABA acid.

D–H···A	D---A(Å)	H---A (Å)	D–H···A(°)	Symmetry codes
C7-H7. . .O6	3.434(1)	2.689(1)	137.61(1)	-x+1,-y,-z+1
O1 -H1. . .O2	3.503(6)	2.900(6)	121.88(1)	-x+1,-y-1,-z
O1-H1. . .O1	3.785(6)	2.831(6)	172.52(1)	-x+1,-y-1,-z
C9-H9A. . .O8	3.531(5)	2.605(5)	162.16(1)	-x+2,-y,-z
C13-H13B. . .O5	3.648(4)	2.779(4)	150.97(1)	-x+2,-y,-z+1
C11-H11A. . .O8	3.7000(3)	2.851(3)	147.92(1)	-x+2,-y,-z+1
C11-H11B. . .O6	3.531(5)	2.605(5)	162.16(1)	-x+1,-y+1,-z+1
C9-H9C. . .O2	3.785(6)	2.831(6)	172.52(1)	x,+y,+z+1

**Fig 4:**
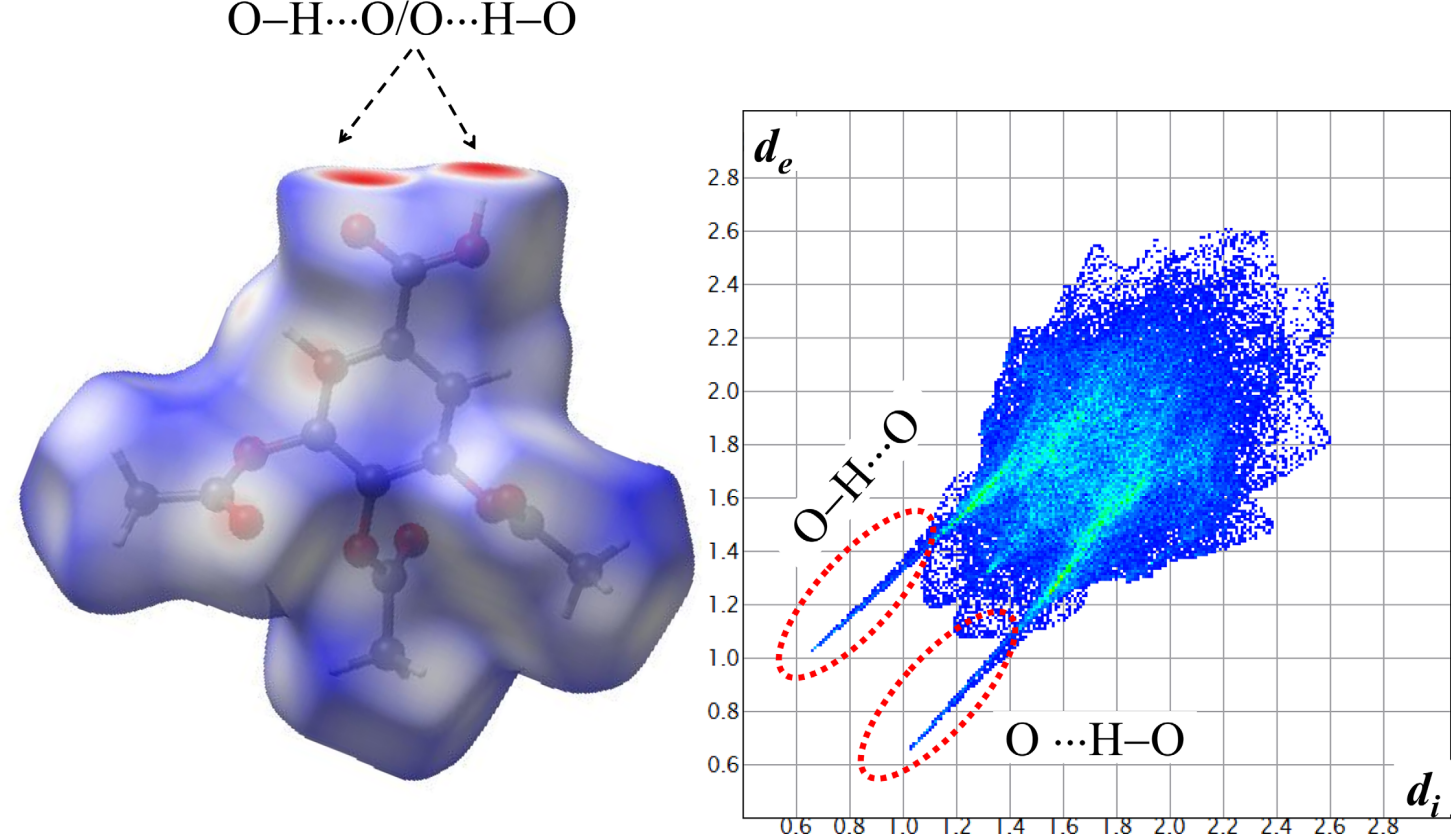
Hirshfeld surface and the 2D fingerprint plots of TABA in the anhydrous forms. The colors represent the number of points that share the same (*d_i_, d_e_*) coordinate (light blue: many; dark blue: few).

The Hirshfeld surface (HS) defines a volume to a molecule in the crystal built on the partitioning of an electron density calculated as the average sum electron density of the spherical atom [38–40]. The fingerprint plots consist in a diagram of the associated *d*_*i*_ *vs*. *d*_*e*_. Thus, this tools summarizes information about intermolecular contacts. Fig 4 shows the Hirshfeld surface and the fingerprint plots of the TABA in the anhydrous form. On the HS, interaction areas (red) located on the O-atom of carboxylic group. From 2D fingerprint, the following features are observed: (*a*) Two long and sharp lateral spikes with minimum (*d*_*i*_ + *d*_*e*_) ≈ 1.8 Å are evident in the diagram. This is the assigned to O−H···O interactions from the $R_{2}^{2}(8)$ homodimer formed by COOH groups (Fig 3, Table 2). These contacts are dominant in the packing of structure corresponding to 43.9% of HS. (*b*) The absence of typical lateral wing at 1.6Ǻ< (*d*_*e*_ + *d*_*i*_) < 2.6Å remarks the inexistence of C−H···π interactions in the structure of anhydrous one (Z' = 1). (*c*) Furthermore, the HS of TABA in the anhydrous form present a great contribution of H···H contacts (33.5% of HS total area) indicating that that structure is also stabilized by *van der Walls* contacts.

**Fig 5:**
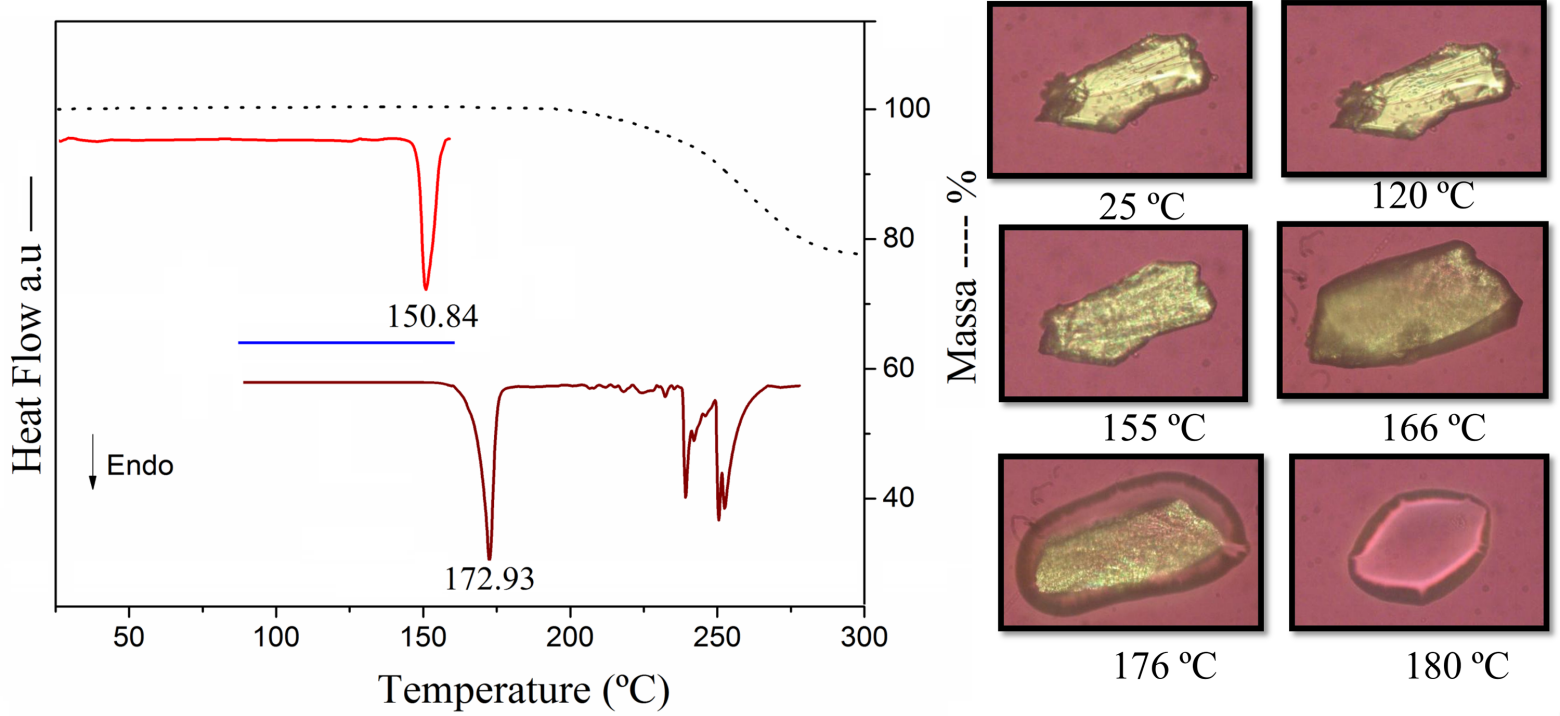
Heating-Cooling-reheating DSC and TGA data for the anhydrous TABA.

With a view to fully characterize the crystalline structure of anhydrous TABA, the DSC/TGA and Hot-stage Microscopy analysis have been performed to this phase and the results are presented in Fig 5. The DSC measurement shows two endothermic event centered at 150.84°C and 172.53°C, respectively. Since no weigh loss is detected in the TGA curve until ~200°C, the first endothermic event is associated to a solid-solid phase transition. This transformation is irreversible, since no corresponding event is occurring during the cooling-reheating cycles of DSC (blue trace, Fig 5). A phase transformation without mass change may occurs due the rearrangement of molecule in the solid state or conformational changes of molecule. Since this solid-solid phase transition does not become single crystals (Fig 5), the SCXR analysis was not possible. Further, the event at 172.53°C in DSC curve corresponds to melting of sample and was confirmed by the Hot-stage analysis. No habit or color change are observed to sample between 25 and 140°C. However, the solid-solid phase transformation is detected in Hot-stage photography when the crystals change their color becoming an opaque material at ~150°C. The melting of sample is achieved at 170°C.

### Optimized structure and Energies

The occurrence of different conformations of the TABA in the anhydrous and hydrated forms provides an opportunity to study the influence of crystal forces on molecular conformations [41–43]. For this purpose, a valuable approach is the comparison of the molecular structure in the solid state with those correspondent ones in the gaseous state, where it is expected that the minimum energy conformation predominates. A search for conformations on the potential surface of TABA have shown several energetic minima, namely TABA-α, TABA-β and TABA-γ (S1 Fig). In order to investigate the relative stability between them, their geometry were fully optimized without constraint at B3LYP/6-311++G(2d,p) level of theory. This hybrid exchange-correlation functional has demonstrated a realistic results for organic molecules [42, 44]. The TABA-β and TABA-γ energies differ from the TABA-α conformer by just 0.249 kcal. mol^-1^ and 3.613 kcal. mol^-1^, respectively. The lowest energy conformation, *i*.*e*. TABA-α, is achieved when the acetoxyl groups are alternately orientated above and below to to main plane of molecule. For TABA dimer full optimized at B97D/6-311++G(2d,p) level in gas phase, the TABA-α geometry is characterized by α = -107.37^0^, β = 96.70^0^ and γ = 112.02^0^ torsion angles and it is remarkably similar to that observed in x-ray data for anhydrous form (S1, S2, S3, S4, S5, S6, S7, S8 and S9 Tables; and also S1 Fig). Otherwise, the higher energy minimum TABA-β (α = -123.80^0^, β = -119.60^0^ and γ = -122.22^0^) and TABA-γ (α = -69.88^0^, β = -70.78^0^, and γ = -120.37^0^), although different, present no alternance of aceotixyl orientations

S1 Table shows a comparison between the geometric parameters of experimental (x-ray data) and calculated structures. The TABA-α monomer conformation optimized at B3LYP/6-311++G(2d,p) has a very good agreement with x-ray data with mean absolute error of 0.014°Å for the bond and 1.17° for the bond angles. The main disagreement values arise in the COOH group. The calculated C1−O1 bond is 6.2% greater than the correspondent in the x-ray data and C1−O2, which it is a double bond, the situation is inverse, *i*.*e*., the x-ray value is 3.5% greater than the calculated. Also, the C2−C1−O1 and C2−C1−O2 calculated bond angles differ significantly from experimental data. The first one is larger than x-ray value in about 3%, while the theoretical value for second angle is smaller than x-ray in 4.4%. As shown in Fig 5, the calculated torsion angles that encompassing the acetoxyl groups are the ones that most diverge from the X-ray data. However, the TABA-α dimer optimized at B97D/6-311++G(2d,p) level, as seen in S1 Table, show results closer to experimental data for COOH group. These results support that the geometric disagreements may be explained by the fact that calculations for the monomer were carried out in gas phase for the monomer and the experimental data were measured in solid state in which the O1 and O2 atoms are involved in strong and dominant hydrogen bonds.

Besides the conformational aspects, the occurrence of anhydrous phase can be also related to stability provided by the homo synthon formed by the COOH groups (graph set: $R_{2}^{2}(8)$, section 3.1). Although the lattice energy does not take in account, an important approach is to determine the energetic contribution of $R_{2}^{2}(8)$ homo-synthon (Fig 3) to structure as a whole. The interaction energy obtained at B97D/6-311++G(2d,p) level for the $R_{2}^{2}(8)$ synthon is of -20.99 kcal.mol^-1^. Note that, the $R_{2}^{2}(8)$ dimeric arrangement require only one stable conformation in the anhydrous form while in hydrate one a conformational multiplicity is present. It suggest that, the water molecule provide the energetic balance for the presence of more than one energetic state of TABA in the hydrated crystal structure of TABA acid.

### Infra-red Spectroscopy

**Fig 6:**
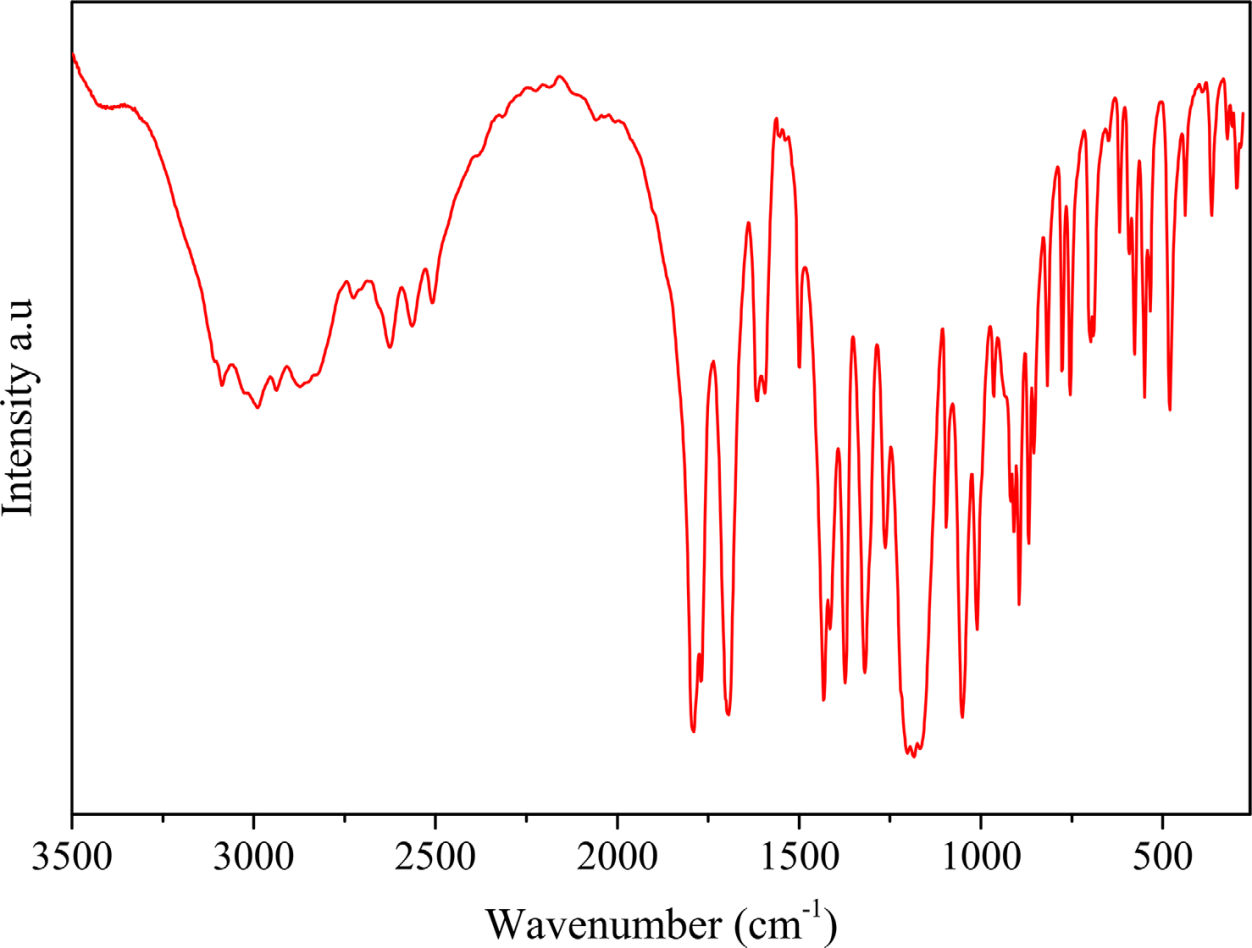
Experimental infrared spectrum of TABA.

Vibrational properties have been shown an important tool for analyzing of structural and supramolecular features of a molecule in the solid state. The experimental and calculated wavenumbers of TABA-α with their respective relative intensities and the correspondent assignments are shown in Table 3. Experimental Spectrum is presented in Fig 6. The calculated frequencies of TABA-α were carried out on the prior fully optimized geometry at B3LYP/6-311++G(2d,p) level of theory, *i*.*e*. TABA-α, and were scaled by 0.9686 factor [45,46] in order to make a better comparison with the experimental values. The Gauss view program package [34] were used to identify atomic displacements corresponding to each vibrational mode. The TABA-α belongs to the *C*_1_ point group symmetry and, as a result, all 93 normal vibrational modes present A symmetries. The calculated IR intensities are important to simplify the assignment of those vibrational modes that are close in frequencies. As shown in Table 3, the calculated frequencies are in good agreement with the experimental values. The disagreements between them are mostly smaller than 25 cm^-1^. This similarity allowed to identify the nature of the vibrational modes. For comparative purpose, the scaled data were used in the follow discussions.

**Table 3:** Calculated and experimental Vibrational spectra for TABA-α. The calculated harmonic frequencies were scaled by a factor of 0.9686.

	B3LYP/6-311++G(2d,p)				
Mode	Nonscaled	Scaled	Intensity	Experimental	assignment
*ν*_1_	21	20	1.22		τ(acetoxy)
*ν*_2_	28	27	0.29		τ(acetoxy)
*ν*_3_	30	29	1.10		τ(acetoxy)
*ν*_4_	43	41	2.27		τ(-CH_3_)
*ν*_5_	50	48	1.20		τ(-CH_3_)
*ν*_6_	53	51	1.70		τ(-CH_3_)
*ν*_7_	55	53	1.40		τ(-COOH)
*ν*_8_	58	56	2.70		τ(-CH_3_)
*ν*_9_	68	66	1.85		τ(-CH_3_)
*ν*_10_	81	78	0.72		τ(-CH_3_)
*ν*_11_	84	82	0.68		τ (-CH_3_)
*ν*_12_	88	85	3.24		τ(-CH_3_)
*ν*_13_	109	106	0.71		γ(ring)
*ν*_14_	150	146	1.52		ω(-COOH) + ω(acetoxy)
*ν*_15_	180	174	2.99	202vw	β(-COOH)
*ν*_16_	205	199	1.25	224m	β(-COOH) + ω(acetoxy)
*ν*_17_	272	264	4.37		τ(acetoxy)
*ν*_18_	274	266	2.52	265vw	π(acetoxy)
*ν*_19_	292	283	2.05		τ(acetoxy)
*ν*_20_	300	291	2.73	298w	τ(acetoxy)
*ν*_21_	315	305	0.26	309vw	τ(acetoxy)
*ν*_22_	342	331	0.11	322vw	τ(acetoxy)
*ν*_23_	386	374	0.74	366w	ω(acetoxy)
*ν*_24_	434	420	13.45		α(acetoxy)
*ν*_25_	456	442	15.46	439vw	α(acetoxy) + β(-C2C1O)
*ν*_26_	525	508	38.87	481m	π(ring) + ω(O-H)
*ν*_27_	538	521	7.08		α(O8C12C13)
*ν*_28_	551	534	11.59	533w	β(ring)
*ν*_29_	563	546	1.77		γ(ring)
*ν*_30_	581	563	7.80	551m	α(acetoxy)
*ν*_31_	593	575	19.51		α(acetoxy) + ω(O-H)
*ν*_32_	596	578	7.24	578w	τ(acetoxy)
*ν*_33_	599	580	34.16		τ(acetoxy)
*ν*_34_	625	606	11.98	592w	ω(C-H) + ω(O-H)
*ν*_35_	651	630	17.07	620w	β(C-H) + β(O-H)
*ν*_36_	666	645	52.68		β(ring) + β(O-H)
*ν*_37_	704	683	9.32	649vw	β(ring) + ν(C-CH3)
*ν*_38_	716	693	4.67	689w	β(ring) + ν(C-CH3)
*ν*_39_	741	718	11.49	690w	ω(ring-H) + β(O-H)
*ν*_40_	785	761	36.91	755w	ω(ring-H) + γ(-COOH)
*ν*_41_	835	809	19.42		θ(ring)
*ν*_42_	856	829	25.00	816w	tw(ring-H) + α(C-C-H)
*ν*_43_	873	845	60.92	854w	ω(ring-H) + α(C-O-C)
*ν*_44_	910	882	49.56	869m	ω(ring-H) + ν(O-COCH3)
*ν*_45_	926	897	5.86	894m	tw(ring-H)
*ν*_46_	943	913	10.34	910m	ω(ring-H)
*ν*_47_	963	933	23.99	919w	β(ring)
*ν*_48_	1003	972	4.75	964vw	α(C-C-H)
*ν*_49_	1017	986	68.35		α(C-C-H)
*ν*_50_	1024	992	78.06		α(C-C-H)
*ν*_51_	1065	1032	221.09	1011m	α(C-C-H)
*ν*_52_	1066	1033	59.01		α(C-C-H)
*ν*_53_	1067	1034	1.76		α(C-C-H)
*ν*_54_	1068	1035	188.99		α(C-C-H)
*ν*_55_	1106	1071	15.80	1051m	β(ring-H)
*ν*_56_	1164	1127	535.87	1095w	β(C-O-H) + β(ring-H)
*ν*_57_	1187	1150	262.35		β(C-O-H) + β(ring-H) + α(C-C-H)
*ν*_58_	1199	1161	719.07	1167s	α(C-C-H)
*ν*_59_	1221	1183	302.67	1186s	ν(O-COCH3) + α(C-C-H)
*ν*_60_	1234	1196	36.30		β(ring-H) + β(ring)
*ν*_61_	1243	1204	5.74	1202s	r(ring-H)
*ν*_62_	1308	1268	89.21	1262w	θ(ring) + β(C-O-H)
*ν*_63_	1332	1291	1.66		ν_symm._(ring)
*ν*_64_	1379	1336	235.09	1320m	β(C-O-H) + θ(ring)
*ν*_65_	1401	1357	27.02		μ(CH3)
*ν*_66_	1402	1358	65.05		μ(CH3)
*ν*_67_	1403	1360	38.53	1373m	μ(CH3)
*ν*_68_	1456	1410	95.71	1415m	ν_asym._(ring)
*ν*_69_	1470	1424	8.48		δ(CH3)
*ν*_70_	1471	1426	30.65	1432s	δ(CH3)
*ν*_71_	1472	1426	1.04		δ(CH3)
*ν*_72_	1476	1430	7.92		δ(CH3)
*ν*_73_	1477	1431	15.04		δ(CH3)
*ν*_74_	1477	1431	3.78		δ(CH3)
*ν*_75_	1519	1472	40.31	1496w	ν_symm._(ring) + β(ring-H)
*ν*_76_	1625	1574	49.54	1556vw	ν_asym._(ring) + β(ring-H)
*ν*_77_	1646	1595	15.98	1594w	ν_symm._(ring) + β(ring-H)
*ν*_78_	1780	1725	383.03	1696s	ν(C = O) of COOH
*ν*_79_	1823	1766	27.50	1767s	ν_symm_ (C = O) of acetoxy
*ν*_80_	1825	1769	131.73		ν_symm_ (C = O) of acetoxy
*ν*_81_	1829	1772	468.55	1793s	ν_asymm_ (C = O) of acetoxy
*ν*_82_	3052	2957	1.18		ν_symm._(CH3)
*ν*_83_	3052	2957	1.10		ν_symm._(CH3)
*ν*_84_	3053	2958	1.23		ν_symm._(CH3)
*ν*_85_	3110	3013	2.91		ν_asymm._(CH3)
*ν*_86_	3110	3014	2.75		ν_asymm._(CH3)
*ν*_87_	3111	3014	3.06		ν_asymm._(CH3)
*ν*_88_	3158	3059	4.87		ν_asymm._(CH3)
*ν*_89_	3158	3060	5.01		ν_asymm._(CH3)
*ν*_90_	3158	3060	4.61		ν_asymm._(CH3)
*ν*_91_	3213	3113	4.26		ν(ring-H)
*ν*_92_	3232	3131	3.00		ν(ring-H)
*ν*_93_	3764	3647	116.82		ν(O-H)

α, angle bending; β, in plane bending; γ, out-of-plane bending; ω, wagging; π, puckering;θ, breathing; δ, scissoring; τ, torsion; r, rocking; ν, stretching; tw, twisting; μ, umbrella movement.

According to our DFT calculations, the bands at 2957, 3013, and 3060 cm^-1^ are assigned to the C−H asymmetric stretching from the methyl groups. The experimental values for these bands are not available in the Table 3. The C−H stretching bands from benzene ring are calculated at 3113 and 3131cm^-1^. These bands differ at 18 cm^-1^ from experimental data. It occurs due to chemical environment differences for these H-atoms. In TABA-α structure, the C7−H7 fragment is associated to O6 atoms by C7−H7···O6 HB while C3−H3 is involved in hydrophobic contacts. The O−H bond-stretching band is computed at 3647cm^-1^ and it display a very intense high relativity intensity.

The bands observed at about at 1767 and 1793 cm^-1^ are assigned to symmetric stretching of C = O from acetoxyl groups. These bands are very intense in the IR spectra and theirs values are very close to the theoretically data. Furthermore, the intense band at 1696 cm^-1^ is ascribed to the C = O stretching of the–COOH group. The classical $R_{2}^{2}(8)$ motif formed by acid-acid association turns the vibration of C = O group different from those related to acetoxyl groups.

The wavenumbers observed at 1496, 1556, 1594 cm^-1^ are mixing modes of symmetric stretching ring and in plane ring-H bending. These bands are weak and their values agree very well with the quantum chemical calculations. The scissoring vibration modes of the methyl groups are attributed to strong band at 1432 cm^-1^. This experimental band split in six computed peaks very close each other (see Table 3). The medium band observed at 1415 cm^-1^ is ascribed mainly to C = C asymmetric stretching of the benzene ring. The umbrella movement of the methyl groups give rise to a medium peak at 1373 cm^-1^. This observed band is also split in three computed peaks.

At 1320 cm^-1^, the medium band observed is mostly attributed to plane bending of C−O−H. The band observed at 1262 cm^-1^ appears in the calculated IR spectra as a weak band and is mainly assigned to benzene ring breathing. The strong peaks at band 1167−1186 cm^-1^ are mostly ascribed to angle deformation modes of C−C−H. A mixing mode of C−O−H in plane bending and ring-H in plane bending give rise to two medium/weak peaks at 1051 and 1095 cm^-1^. The C−C−H angle bending give rise to a broad peak with medium intensity at 1011 cm^-1^. The ring−H wagging and ring−H twisting vibrational modes display medium peaks at 816, 854, 869, 894, 910, and 919 cm^-1^ in the infrared spectra of the TABA.

The peaks observed in the 592−755 cm^-1^ region are of weak intensities and they are mixing modes of C−H wagging, in plane ring deformation, C−CH_3_ stretching and ring−H wagging. The acetoxyl angle bending give rise to band at 551 cm^-1^ and the torsion mode of acetoxyl give rise to a band at 578 cm^-1^. Several weak peaks are observed in 265–439 cm^-1^ region and they are mainly assigned to the torsion of the acetoxyl groups.

### Frontier molecular orbitals and molecular electrostatic potential (MEP)

**Fig 7:**
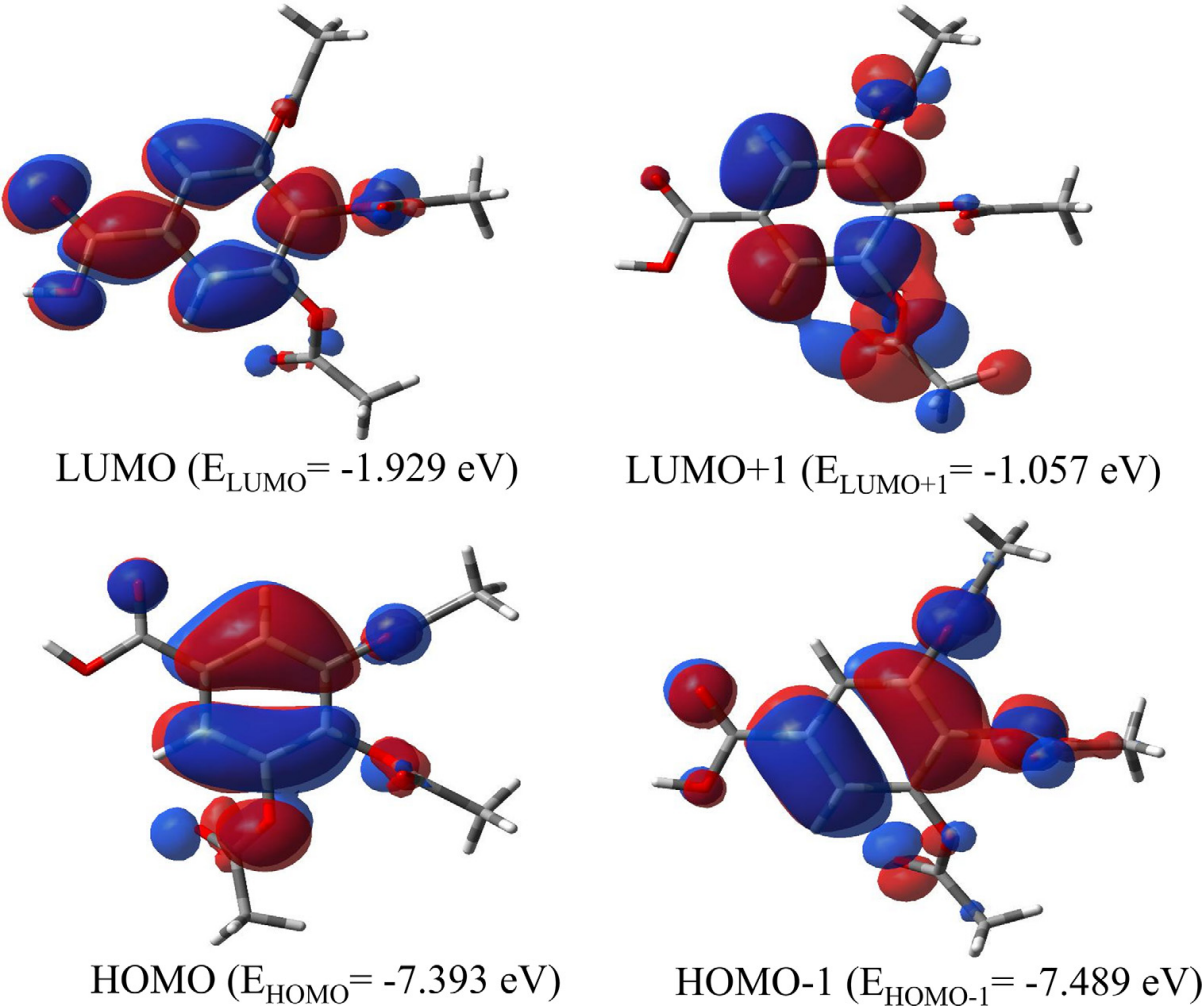
Isodensity surface of HOMO, HOMO-1, LUMO and LUMO+1 for TABA in gas phase. The calculated energy gap (Egap = E_LUMO_ - E_HOMO_) at B3LYP/6-311++G(2d,p) level is of 5.464 eV.

Because the GA derivatives have appreciable antioxidant properties, it is important to characterize the nature of TABA-α in terms of chemical reactivity. Usually, the study of frontier orbitals is a useful to this purpose. Fig 7 shows a graphical representation of the HOMO, HOMO-1, LUMO and LUMO+1 frontier molecular orbitals. Both HOMO and HOMO-1 orbitals present π bonding symmetries and are situated predominantly on the aromatic ring. The LUMO and LUMO+1 molecular orbitals are both π* antibonding symmetries and they are spread out mainly on the aromatic ring. All oxygen atoms contribute significantly with *p* orbitals in the formation of the LUMO-1. However, the contribution of the *p* orbitals from the oxygen atoms in the HOMO orbital formation is lower than that observed in the HOMO-1. It is also observed that the oxygen of the OH fragment has no contribution in the HOMO orbital formation. The *p* orbitals from the oxygen atoms also contribute for the LUMO and LUMO+1 formations. The LUMO+1 has a higher contribution of the *p* orbitals from the oxygen of the acetoxyl groups, while the oxygen atoms from the carbonyl and hydroxyl groups contribute markedly in the LUMO formation. The LUMO energy of -1.929 eV shows that the TABA-α acid is a good electrophile and the energy gap (HOMO—LUMO) of 5.464 eV shows that this molecule also has a very high chemical stability.

**Fig 8:**
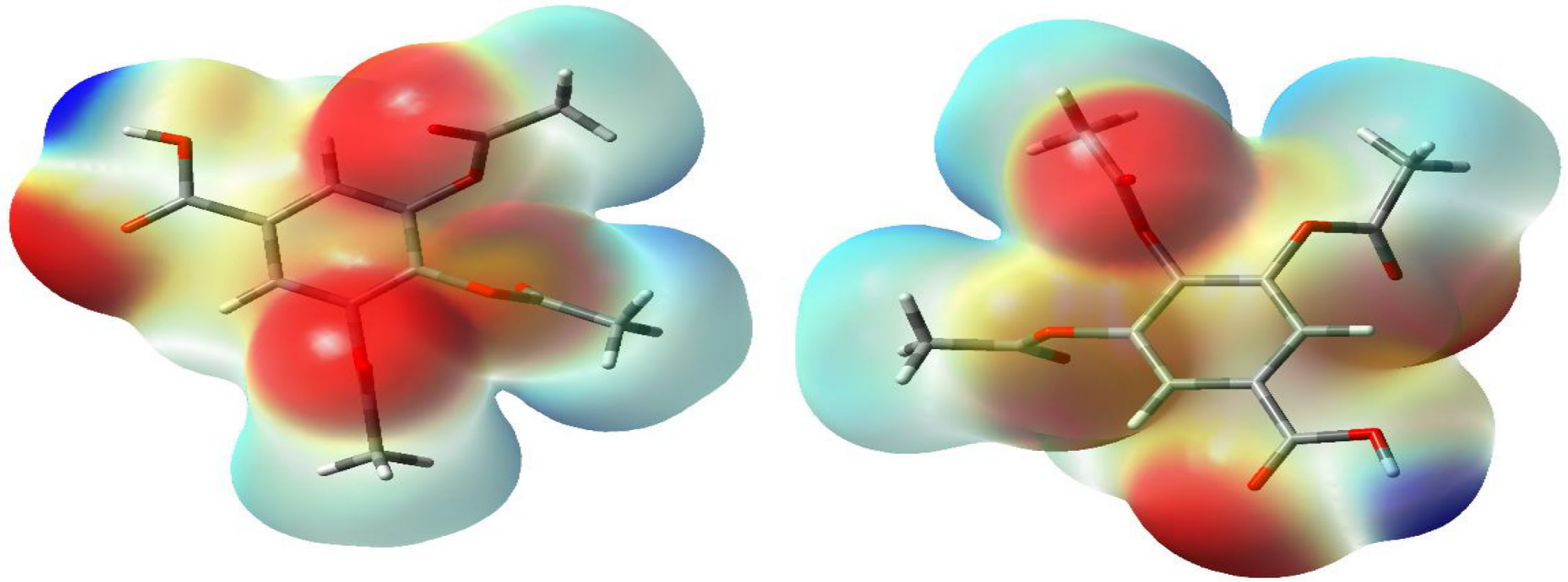
Two different views of the calculated molecular electrostatic potential surface *ρ*(*r*) = 4.0 × 10^−4^ electrons/bohr^3^ contour of the total electron density of TABA. The red color stands for regions where the electrostatic potential is negative and blue color stands for positive regions.

Fig 8 shows a graphical representation of the MEP of TABA-α computed at *ρ*(***r***) = 4.0 × 10^−4^ electrons/bohr^3^ contour encompassing the molecule. The positive and negative values on the MEP surface correspond to electron density is locally depleted and locally concentrated, respectively. The minima (the most negative values) on the MEP surface is observed on the oxygen atoms and they represent regions of higher electron density and, in turn, sites for nucleophilic attacked. On the O-atoms from COOH group, the MEP energy is of about -27 kcal/mol, while the O-atoms from acetoxyl group are about -11 kcal/mol. On the aromatic ring, the MEP energy is of about -5 kcal/mol. Based on this interpretation, the oxygen sites of TABA acid susceptible to nucleophilic attack can be ranked as follow: C = O > −O− > aromatic ring

The maximum on the MEP surface occur on hydrogen atom from COOH group in which the MEP energy is of about 33 kcal/mol. This large positive value (blue color) indicate that this region is relatively depleted in electrons density and this site tend to attract chemical species that are rich in electrons. Another maximum region is remarked to H-aromatic and methyl hydrogen atoms (11 kcal/mol and 16 kcal/mol, respectively). Considering the depletion of the electron density, the hydrogen atoms of the TABA-α acid can also be ranked, from the most depleted to the least depleted, as follow: O−H > H−CH_3_− > H−aromatic.

## Concluding Remarks

Anhydrous structure of 3,4,5-triacetoxibenzoic acid (TABA) was investigated by a combined experimental and theoretical analysis using X-ray diffractions, FT-IR techniques and DFT calculations. A detailed supramolecular analysis of anhydrous TABA reveals that the acid-acid supramolecular motif is the main build block of structure. The thermal analysis shows an irreversible solid-solid transition phase at 155°C. This event is evident in HSM images. This transition occurs generating an additional anhydrous TABA phase. Conformational searches at B3LYP/6-311++G(2d,p) level of theory show that exist three stable conformations. The most stable conformation resulting from the conformational analysis is the conformation observed experimentally in its anhydrous form. Furthermore, X-ray data agree very well with the geometrical parameters obtained from theoretical calculation. The reactivity of TABA was investigated by analysis of the Frontier molecular orbitals and MEP surface. These analyses show that the oxygen sites of TABA are sites of nucleophilic attack and they can be ranked as C = O > −O− > aromatic ring. There is a good agreement between experimental and calculated infrared spectra allowing the assignment of the vibrational normal modes. The present investigation advances the understanding of the structural and supramolecular features of TABA, which is important for biological applicability.

## Supporting Information

S1 Fig The three possible geometric configurations for TABA acid: (a) TABA-α, (b) TABA-β and (c) TABA-γ. The calculation results at B3LYP/6-311++G(2d,p) level show that TABA-α is the most stable configuration, while TABA-β and TABA-γ conformations were computed as 0.249 kcal/mol^-1^ and 3.613 kcal/mol^-1^, respectively, at B3LYP/6-311++G(2d,p) level of theory.
(TIFF)

S1 Table Geometric parameters by X-ray for TABA and theoretical calculations at B3LYP/6-311++G(2d,p) and B97D/6-311++G(2d,p) level of theory for the monomer and dimer, respectively. The bond lengths are given in angstroms and the bond angles and dihedral angles are given in degrees. All the calculations were carried out at gas phase.
(DOCX)

S2 Table Crystal data and structure refinement for the TABA. (DOCX)

S3 Table Fractional Atomic Coordinates (×10^4^) and Equivalent Isotropic Displacement Parameters (Å^2^×10^3^) for shelxl. U_eq_ is defined as 1/3 of of the trace of the orthogonalised U_IJ_ tensor.
(DOCX)

S4 Table Anisotropic Displacement Parameters (Å^2^×10^3^) for shelxl. The Anisotropic displacement factor exponent takes the form: -2π^2^[h^2^a*^2^U_11_+2hka*b*U_12_+…].
(DOCX)

S5 Table Bond Lengths for TABA. (DOCX)

S6 Table Bond Angles for TABA. (DOCX)

S7 Table Torsion Angles for TABA. (DOCX)

S8 Table Hydrogen Atom Coordinates (Å×10^4^) and Isotropic Displacement Parameters (Å^2^×10^3^) for TABA. (DOCX)

S9 Table Check-Cif report for TABA. (DOCX)
