# Supplementary figures and images for: Structural and Theoretical Investigation of Anhydrous 3,4,5-Triacetoxybenzoic Acid
Source: PLoS One. 2016 Jun 29;11(6):e0158029. doi: 10.1371/journal.pone.0158029 (PMC4927074; doi:10.1371/journal.pone.0158029)

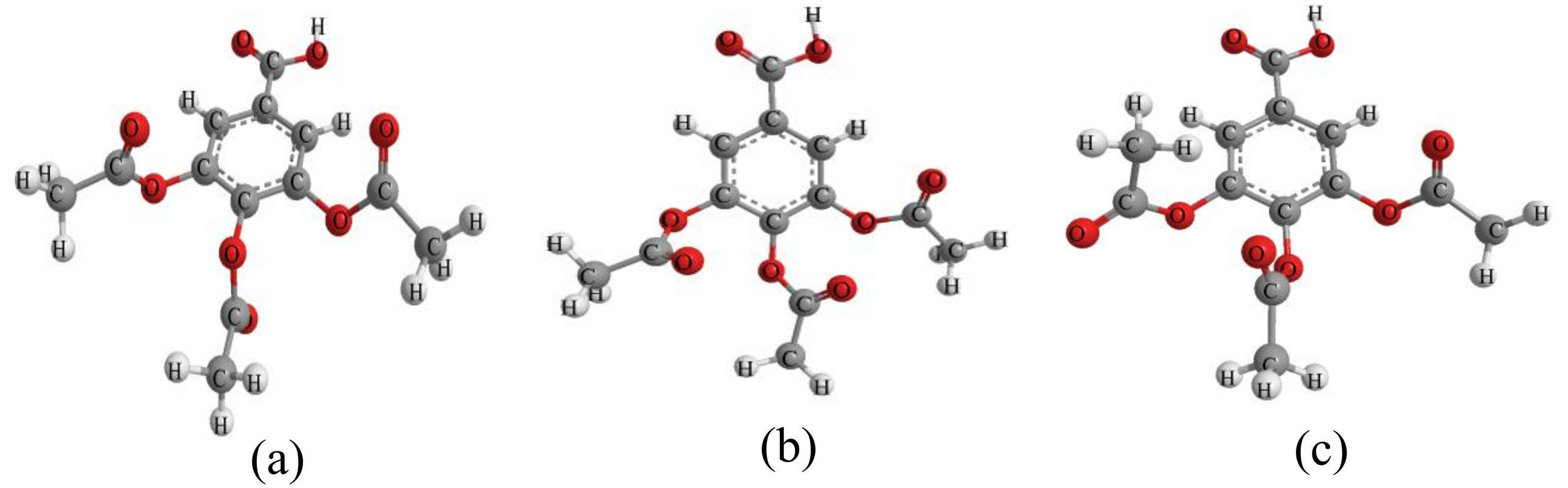

Supplement: S1 Fig — The calculation results at B3LYP/6-311++G(2d,p) level show that TABA-α is the most stable configuration, while TABA-β and TABA-γ conformations were computed as 0.249 kcal/mol-1 and 3.613 kcal/mol-1, respectively, at B3LYP/6-311++G(2d,p) level of theory. (TIFF) [file pone.0158029.s001.tiff]
