# Supplementary material for: Structural and Theoretical Investigation of Anhydrous 3,4,5-Triacetoxybenzoic Acid
Source: PLoS One. 2016 Jun 29;11(6):e0158029. doi: 10.1371/journal.pone.0158029 (PMC4927074; doi:10.1371/journal.pone.0158029)
Supplement: S1 Table — The bond lengths are given in angstroms and the bond angles and dihedral angles are given in degrees. All the calculations were carried out at gas phase. (DOCX) [file pone.0158029.s002.docx]

**S1 Table**. Geometric parameters by X-ray for TABA and theoretical calculations at B3LYP/6-311++G(2d,p) and B97D/6-311++G(2d,p) level of theory for the monomer and dimer, respectively. The bond lengths are given in angstroms and the bond angles and dihedral angles are given in degrees. All the calculations were carried out at gas phase.

| **Bond length** | **Exp.** | **Calculated** | | **Bond angle** | **Exp.** | **Calculated** | |
| --- | --- | --- | --- | --- | --- | --- | --- |
|  |  | **Monomer**  **B3LYP** | **Dimer**  **B97D** |  |  | **Monomer**  **B3LYP** | **Dimer**  **B97D** |
| C(1)−C(2) | 1.487 | 1.486 | 1.491 | C(2)−C(1)−O(1) | 116.76 | 113.08 | 114.53 |
| C(1)−O(1) | 1.276 | 1.355 | 1.325 | C(2)−C(1)−O(2) | 119.15 | 124.67 | 121.61 |
| C(1)−O(2) | 1.251 | 1.207 | 1.238 | C(2)−C(3)−C(4) | 120.33 | 119.57 | 119.66 |
| C(2)−C(3) | 1.382 | 1.395 | 1.404 | C(3)−C(4)−C(5) | 120.40 | 120.59 | 120.66 |
| C(2)−C(7) | 1.392 | 1.394 | 1.403 | C(4)−C(5)−C(6) | 118.76 | 119.46 | 119.29 |
| C(3)−C(4) | 1.372 | 1.380 | 1.387 | C(5)−C(6)−C(7) | 121.53 | 120.57 | 120.76 |
| C(4)−C(5) | 1.386 | 1.392 | 1.400 | C(6)−C(7)−C(2) | 118.67 | 119.39 | 119.49 |
| C(4)−O(3) | 1.396 | 1.389 | 1.393 | C(7)−C(2)−C(3) | 120.24 | 120.41 | 120.14 |
| C(5)−C(6) | 1.388 | 1.392 | 1.398 | C(4)−O(3)−C(8) | 117.79 | 117.61 | 117.38 |
| C(5)−O(5) | 1.384 | 1.380 | 1.384 | O(3)−C(8)−O(4) | 122.36 | 123.26 | 123.73 |
| C(6)−C(7) | 1.377 | 1.385 | 1.390 | O(3)−C(8)−C(9) | 110.25 | 109.98 | 109.19 |
| C(6)−O(7) | 1.394 | 1.386 | 1.392 | C(5)−O(5)−C(10) | 116.74 | 117.54 | 117.03 |
| C(8)−O(3) | 1.363 | 1.379 | 1.393 | O(5)−C(10)−O(6) | 122.05 | 123.18 | 123.61 |
| C(8)−O(4) | 1.197 | 1.196 | 1.202 | O(5)−C(10)−C(11) | 109.96 | 109.90 | 109.14 |
| C(8)−C(9) | 1.493 | 1.502 | 1.510 | C(6)−O(7)−C(12) | 117.10 | 119.15 | 117.87 |
| C(10)−O(5) | 1.381 | 1.382 | 1.395 | O(7)−C(12)−O(8) | 122.31 | 123.64 | 123.88 |
| C(10)−O(6) | 1.192 | 1.195 | 1.202 | O(7)−C(12)−C(13) | 110.25 | 109.75 | 109.07 |
| C(10)−C(11) | 1.478 | 1.501 | 1.509 | C(3)−C(2)−C(1) −O(2) | 4.25 | 0.20 | -0.53 |
| C(12)−O(7) | 1.365 | 1.380 | 1.394 | C(3)−C(2)−C(1) −O(1) | -175.92 | -179.69 | 179.39 |
| C(12)−O(8) | 1.192 | 1.196 | 1.202 | C(4)−C(3)−C(2) −C(1) | -179.85 | 179.70 | -179.94 |
| C(12)−C(13) | 1.483 | 1.502 | 1.510 | C(5)−C(4)−C(3) −C(2) | 1.23 | 0.22 | -0.68 |
| O(1)−H | 0.820 | 0.980 | 1.016 | C(2)−C(3)−C(4) −O(3) | -176.16 | -176.10 | 175.81 |
| C(3)−H | 0.930 | 1.081 | 1.086 | C(3)−C(4)−O(3) −C(8) | -109.23 | -100.26 | 107.38 |
| C(7)−H | 0.930 | 1.079 | 1.084 | C(4)−O(3)−C(8) −O(4) | -11.47 | -0.17 | -2.91 |
|  |  |  |  | C(4)−O(3)−C(8) −C(9) | 169.53 | 179.26 | 177.42 |
|  |  |  |  | C(4)−C(5)−O(5) −C(10) | 118.38 | 98.49 | -96.69 |
|  |  |  |  | C(5)−O(5)−C(10) −O(6) | -14.27 | -1.98 | 2.227 |
|  |  |  |  | C(5)−O(5)−C(10) −C(11) | 165.64 | 178.08 | -177.65 |
|  |  |  |  | C(4)−C(5)−C(6) −O(7) | 179.91 | -176.44 | 175.56 |
|  |  |  |  | C(5)−C(6)−O(7) −C(12) | 112.99 | -122.11 | 112.02 |
|  |  |  |  | C(6)−O(7)−C(12) −O(8) | 4.07 | 0.25 | 0.10 |
|  |  |  |  | C(6)−O(7)−C(12) −C(13) | -175.75 | -179.80 | -179.85 |
