# Supplementary material for: Structural and Theoretical Investigation of Anhydrous 3,4,5-Triacetoxybenzoic Acid
Source: PLoS One. 2016 Jun 29;11(6):e0158029. doi: 10.1371/journal.pone.0158029 (PMC4927074; doi:10.1371/journal.pone.0158029)
Supplement: S2 Table — (DOCX) [file pone.0158029.s003.docx]

**S2 Table.** Crystal data and structure refinement for the TABA.

| Empirical formula | C_13_H_12_O_8_ |
| --- | --- |
| Formula weight | 296.23 |
| Crystal system | Triclinic |
| Space group | $P\bar{1}$ |
| a/Å | 8.3990(11) |
| b/Å | 8.4870(8) |
| c/Å | 9.8050(11) |
| α/° | 87.999(7) |
| β/° | 82.508(6) |
| γ/° | 88.714(7) |
| Volume/Å^3^ | 692.42(14) |
| Z | 2 |
| ρ_calc_/g.cm^-3^ | 1.421 |
| μ/mm^‑1^ | 0.120 |
| F(000) | 308.0 |
| Radiation | MoKα (λ = 0.71073) |
| 2θ range for data collection/º | 6.274 to 53.48 |
| Reflections collected | 2915 |
| Independent reflections | 2915 [R_int_ = 0.038] |
| Data/restraints/parameters | 2915/0/193 |
| Goodness-of-fit on F^2^ | 1.028 |
| Final R indexes [I > 2σ (I)] | R_1_ = 0.0479, wR_2_ = 0.1313 |
| Final R indexes [all data] | R_1_ = 0.0621, wR_2_ = 0.1417 |
| Largest diff. peak/hole / e Å^-3^ | 0.30/-0.23 |
