# Supplementary material for: Structural and Theoretical Investigation of Anhydrous 3,4,5-Triacetoxybenzoic Acid
Source: PLoS One. 2016 Jun 29;11(6):e0158029. doi: 10.1371/journal.pone.0158029 (PMC4927074; doi:10.1371/journal.pone.0158029)
Supplement: S3 Table — Ueq is defined as 1/3 of of the trace of the orthogonalised UIJ tensor. (DOCX) [file pone.0158029.s004.docx]

**S3 Table.** Fractional Atomic Coordinates (×10^4^) and Equivalent Isotropic Displacement Parameters (Å^2^×10^3^) for shelxl. U_eq_ is defined as 1/3 of of the trace of the orthogonalised U_IJ_ tensor.

| **Atom** | ***x*** | ***y*** | ***z*** | **U(eq)** |
| --- | --- | --- | --- | --- |
| O5 | 7828.4(13) | 2384.3(12) | 2950.5(11) | 45.9(3) |
| O7 | 6942.6(14) | -126.0(13) | 4681.7(10) | 46.5(3) |
| C2 | 6291.1(18) | -1712.7(18) | 1368.4(15) | 41.8(4) |
| O3 | 7553.5(13) | 2213.5(14) | 108.2(12) | 51.0(3) |
| O6 | 5351.7(15) | 2946.1(14) | 4020.0(14) | 59.4(4) |
| C4 | 7210.7(17) | 932.8(18) | 1023.6(15) | 40.8(3) |
| C6 | 6865.5(18) | -266.7(18) | 3279.7(14) | 41.2(4) |
| C5 | 7274.3(17) | 1039.3(17) | 2423.6(15) | 39.7(3) |
| C7 | 6341.2(19) | -1630.7(18) | 2777.6(15) | 42.6(4) |
| O2 | 5872.2(15) | -3264.2(15) | -504.5(12) | 56.9(3) |
| O1 | 5295(2) | -4292.7(15) | 1622.7(14) | 73.5(4) |
| C12 | 8085(2) | -1014(2) | 5238.2(16) | 48.9(4) |
| C1 | 5787.7(19) | -3181.9(19) | 774.0(16) | 46.0(4) |
| O8 | 9044.1(18) | -1813.8(19) | 4556.5(15) | 74.5(4) |
| C10 | 6756(2) | 3191.9(17) | 3876.5(16) | 45.4(4) |
| C3 | 6738.2(18) | -434.6(19) | 501.1(15) | 42.4(4) |
| O4 | 10151.8(17) | 2117(2) | 431.0(17) | 79.3(5) |
| C8 | 9118(2) | 2637(2) | -199.6(19) | 56.1(5) |
| C11 | 7621(3) | 4334(2) | 4601(2) | 67.0(5) |
| C9 | 9295(3) | 3815(3) | -1375(2) | 82.8(7) |
| C13 | 7927(3) | -820(3) | 6748.0(18) | 69.8(6) |
