# Supplementary material for: Structural and Theoretical Investigation of Anhydrous 3,4,5-Triacetoxybenzoic Acid
Source: PLoS One. 2016 Jun 29;11(6):e0158029. doi: 10.1371/journal.pone.0158029 (PMC4927074; doi:10.1371/journal.pone.0158029)
Supplement: S4 Table — The Anisotropic displacement factor exponent takes the form: -2π2[h2a*2U11+2hka*b*U12+…]. (DOCX) [file pone.0158029.s005.docx]

**S4 Table.** Anisotropic Displacement Parameters (Å^2^×10^3^) for shelxl. The Anisotropic displacement factor exponent takes the form: -2π^2^[h^2^a*^2^U_11_+2hka*b*U_12_+…].

| **Atom** | **U_11_** | **U_22_** | **U_33_** | **U_23_** | **U_13_** | **U_12_** |
| --- | --- | --- | --- | --- | --- | --- |
| O5 | 47.2(6) | 45.8(6) | 43.9(6) | -11.5(5) | 1.0(5) | -5.0(5) |
| O7 | 60.4(7) | 49.8(6) | 30.3(5) | -7.3(4) | -8.1(5) | 2.1(5) |
| C2 | 44.0(8) | 45.6(8) | 37.1(8) | -8.1(6) | -9.7(6) | 3.6(6) |
| O3 | 49.3(7) | 57.5(7) | 45.0(6) | 9.2(5) | -3.7(5) | -4.6(5) |
| O6 | 53.6(8) | 53.5(7) | 68.1(8) | -9.0(6) | 4.0(6) | 4.6(5) |
| C4 | 39.6(8) | 46.9(8) | 34.8(7) | 0.4(6) | -2.4(6) | 2.3(6) |
| C6 | 46.9(8) | 47.1(8) | 30.1(7) | -5.7(6) | -6.4(6) | 3.5(6) |
| C5 | 39.8(8) | 40.8(8) | 38.6(8) | -7.6(6) | -4.3(6) | 1.5(6) |
| C7 | 51.5(9) | 40.7(8) | 36.1(8) | -3.6(6) | -7.2(6) | 2.0(6) |
| O2 | 65.3(8) | 64.8(8) | 42.7(6) | -17.0(5) | -9.8(5) | -7.1(6) |
| O1 | 122.4(13) | 48.6(7) | 54.6(8) | -4.3(6) | -26.8(8) | -20.7(7) |
| C12 | 55.5(10) | 53.2(9) | 39.6(8) | -2.1(7) | -11.5(7) | -4.4(7) |
| C1 | 52.9(9) | 46.5(9) | 40.4(8) | -7.0(7) | -12.4(7) | 3.6(7) |
| O8 | 78.3(10) | 89.8(10) | 58.7(8) | -13.7(7) | -23.1(7) | 26.2(8) |
| C10 | 55.1(10) | 37.8(8) | 41.3(8) | -1.9(6) | 0.3(7) | 5.1(7) |
| C3 | 42.8(8) | 53.0(9) | 32.1(7) | -5.5(6) | -6.9(6) | 4.0(6) |
| O4 | 48.7(8) | 102.1(12) | 84.4(11) | 0.9(9) | -0.1(7) | 0.6(7) |
| C8 | 53.8(10) | 60.4(10) | 51.2(10) | -7.1(8) | 7.1(8) | -6.2(8) |
| C11 | 78.9(13) | 55.3(11) | 68.0(12) | -23.0(9) | -8.3(10) | -0.3(9) |
| C9 | 93.1(17) | 86.8(15) | 63.2(13) | 8.6(11) | 11.7(11) | -26.4(13) |
| C13 | 81.3(14) | 92.4(15) | 38.5(9) | 1.6(9) | -17.8(9) | -9.7(11) |
