# Supplementary material for: Structural and Theoretical Investigation of Anhydrous 3,4,5-Triacetoxybenzoic Acid
Source: PLoS One. 2016 Jun 29;11(6):e0158029. doi: 10.1371/journal.pone.0158029 (PMC4927074; doi:10.1371/journal.pone.0158029)
Supplement: S5 Table — (DOCX) [file pone.0158029.s006.docx]

**S5 Table.** Bond Lengths for TABA.

| **Bond** | **Length (Å)** |  | **Bond** | | **Length (Å)** |
| --- | --- | --- | --- | --- | --- |
| O5−C5 | 1.383(2) |  | C4−C3 | | 1.372(2) |
| O5−C10 | 1.381(2) |  | C6−C5 | | 1.389(2) |
| O7−C6 | 1.394(2) |  | C6−C7 | | 1.377(2) |
| O7−C12 | 1.365(2) |  | O2−C1 | | 1.251(2) |
| C2−C7 | 1.392(2) |  | O1−C1 | | 1.275(2) |
| C2−C1 | 1.487(2) |  | C12−O8 | | 1.193(2) |
| C2−C3 | 1.383(2) |  | C12−C13 | | 1.483(2) |
| O3−C4 | 1.395(2) |  | C10−C11 | | 1.479(2) |
| O3−C8 | 1.362(2) |  | O4−C8 | | 1.198(2) |
| O6−C10 | 1.192(2) |  | C8−C9 | | 1.494(3) |
| C4−C5 | 1.387(2) |  |  |  |  |
