# Supplementary material for: Structural and Theoretical Investigation of Anhydrous 3,4,5-Triacetoxybenzoic Acid
Source: PLoS One. 2016 Jun 29;11(6):e0158029. doi: 10.1371/journal.pone.0158029 (PMC4927074; doi:10.1371/journal.pone.0158029)
Supplement: S6 Table — (DOCX) [file pone.0158029.s007.docx]

**S6 Table**. Bond Angles for TABA.

| **Bond angle** | **Angle (˚)** |  | **Bond angle** | **Angle (˚)** |
| --- | --- | --- | --- | --- |
| C10−O5−C5 | 116.72(12) |  | C6−C7−C2 | 118.70(15) |
| C12−O7−C6 | 117.10(12) |  | O7−C12−C13 | 110.30(15) |
| C7−C2−C1 | 120.56(14) |  | O8−C12−O7 | 122.30(15) |
| C3−C2−C7 | 120.23(14) |  | O8−C12−C13 | 127.40(17) |
| C3−C2−C1 | 119.20(13) |  | O2−C1−C2 | 119.15(15) |
| C8−O3−C4 | 117.79(13) |  | O2−C1−O1 | 124.06(15) |
| C5−C4−O3 | 121.87(14) |  | O1−C1−C2 | 116.79(14) |
| C3−C4−O3 | 117.66(13) |  | O5−C10−C11 | 109.94(15) |
| C3−C4−C5 | 120.42(14) |  | O6−C10−O5 | 122.07(15) |
| C5−C6−O7 | 117.46(13) |  | O6−C10−C11 | 128.00(16) |
| C7−C6−O7 | 120.95(14) |  | C4−C3−C2 | 120.32(14) |
| C7−C6−C5 | 121.54(13) |  | O3−C8−C9 | 110.28(18) |
| O5−C5−C4 | 120.81(13) |  | O4−C8−O3 | 122.38(17) |
| O5−C5−C6 | 120.34(13) |  | O4−C8−C9 | 127.33(19) |
| C4−C5−C6 | 118.73(13) |  |  |  |
