# Supplementary material for: Structural and Theoretical Investigation of Anhydrous 3,4,5-Triacetoxybenzoic Acid
Source: PLoS One. 2016 Jun 29;11(6):e0158029. doi: 10.1371/journal.pone.0158029 (PMC4927074; doi:10.1371/journal.pone.0158029)
Supplement: S7 Table — (DOCX) [file pone.0158029.s008.docx]

**S7 Table**. Torsion Angles for TABA.

| **Torsion angle** | **Angle (˚)** |  | **Torsion angle** | **Angle (˚)** |
| --- | --- | --- | --- | --- |
| O7−C6−C5−O5 | 3.9(2) |  | C7−C2−C3−C4 | -1.0(2) |
| O7−C6−C5−C4 | 179.92(13) |  | C7−C6−C5−O5 | -178.70(13) |
| O7−C6−C7−C2 | -179.82(13) |  | C7−C6−C5−C4 | -2.6(2) |
| O3−C4−C5−O5 | -6.1(2) |  | C12−O7−C6−C5 | -113.02(16) |
| O3−C4−C5−C6 | 177.85(13) |  | C12−O7−C6−C7 | 69.52(19) |
| O3−C4−C3−C2 | -176.17(13) |  | C1−C2−C7−C6 | 177.84(14) |
| C4−O3−C8−O4 | -11.5(3) |  | C1−C2−C3−C4 | -179.86(13) |
| C4−O3−C8−C9 | 169.50(16) |  | C10−O5−C5−C4 | 118.39(15) |
| C6−O7−C12−O8 | 4.1(2) |  | C10−O5−C5−C6 | -65.63(18) |
| C6−O7−C12−C13 | -175.72(15) |  | C3−C2−C7−C6 | -1.0(2) |
| C5−O5−C10−O6 | -14.3(2) |  | C3−C2−C1−O2 | 4.3(2) |
| C5−O5−C10−C11 | 165.67(14) |  | C3−C2−C1−O1 | -175.91(15) |
| C5−C4−C3−C2 | 1.2(2) |  | C3−C4−C5−O5 | 176.62(13) |
| C5−C6−C7−C2 | 2.8(2) |  | C3−C4−C5−C6 | 0.6(2) |
| C7−C2−C1−O2 | -174.53(15) |  | C8−O3−C4−C5 | 73.39(19) |
| C7−C2−C1−O1 | 5.3(2) |  | C8−O3−C4−C3 | -109.26(16) |
