# Supplementary material for: Structural and Theoretical Investigation of Anhydrous 3,4,5-Triacetoxybenzoic Acid
Source: PLoS One. 2016 Jun 29;11(6):e0158029. doi: 10.1371/journal.pone.0158029 (PMC4927074; doi:10.1371/journal.pone.0158029)
Supplement: S8 Table — (DOCX) [file pone.0158029.s009.docx]

**S8 Table**. Hydrogen Atom Coordinates (Å×10^4^) and Isotropic Displacement Parameters (Å^2^×10^3^) for TABA.,

| **Atom** | ***x*** | ***y*** | ***z*** | **U(eq)** |
| --- | --- | --- | --- | --- |
| H7 | 6027 | -2480 | 3368 | 51 |
| H1 | 4819 | -5099 | 1303 | 69 |
| H3 | 6719 | -502 | -441 | 51 |
| H11A | 8316 | 3775 | 5163 | 101 |
| H11B | 6860 | 4965 | 5173 | 101 |
| H11C | 8250 | 5003 | 3939 | 101 |
| H9A | 9251 | 3288 | -2220 | 124 |
| H9B | 10308 | 4328 | -1413 | 124 |
| H9C | 8440 | 4587 | -1248 | 124 |
| H13A | 7913 | 282 | 6940 | 105 |
| H13B | 8820 | -1334 | 7109 | 105 |
| H13C | 6945 | -1284 | 7172 | 105 |
