# Supplementary material for: Structural and Theoretical Investigation of Anhydrous 3,4,5-Triacetoxybenzoic Acid
Source: PLoS One. 2016 Jun 29;11(6):e0158029. doi: 10.1371/journal.pone.0158029 (PMC4927074; doi:10.1371/journal.pone.0158029)
Supplement: S9 Table — (DOCX) [file pone.0158029.s010.docx]

**S9 Table.** Check-Cif report for TABA

| Bond precision: | C-C = 0.0022 Å | |
| --- | --- | --- |
| Wavelength | 0.71073 | |
| Temperature | 298 K | |
| Cell dimensions | a = 8.3990(11) **Å**  b = 8.4870(8) **Å**  c = 9.8050(11) **Å**  alpha = 87.999(7)**˚**  beta = 82.508(6)**˚**  gamma = 88.714(7)**˚** | |
| Data completeness | 0.987 | |
| Theta(max) | 26.740 | |
| R(reflections)= | 0.0479( 2291) | |
| wR2(reflections)= | 0.1416( 2915) | |
|  | **Calculated** | **Reported** |
| Volume | 692.42(14) | 692.42(14) |
| Space group | P -1 | P -1 |
| Hall group | -P 1 | -P 1 |
| Moiety formula | C13 H12 O8 | C13 H12 O8 |
| Sum formula | C13 H12 O8 | C13 H12 O8 |
| Mr | 296.23 | 296.23 |
| Dx,g cm-3 | 1.421 | 1.421 |
| Z | 2 | 2 |
| Mu (mm-1) | 0.120 | 0.120 |
| F000 | 308.0 | 308.0 |

The following ALERTS were generated. Each ALERT has the format

**test-name_ALERT_alert-type_alert-level**.

Click on the hyperlinks for more details of the test.

**Alert level C**

[PLAT906_ALERT_3_C](javascript:makeHelpWindow(%22PLAT906.html%22)) Large K value in the Analysis of Variance ...... 2.745 Check

[PLAT911_ALERT_3_C](javascript:makeHelpWindow(%22PLAT911.html%22)) Missing # FCF Refl Between THmin & STh/L= 0.600 26 Report

[PLAT913_ALERT_3_C](javascript:makeHelpWindow(%22PLAT913.html%22)) Missing # of Very Strong Reflections in FCF .... 21 Note

[PLAT978_ALERT_2_C](javascript:makeHelpWindow(%22PLAT978.html%22)) Number C-C Bonds with Positive Residual Density 0 Note

**Alert level G**

[PLAT007_ALERT_5_G](javascript:makeHelpWindow(%22PLAT007.html%22)) Number of Unrefined Donor-H Atoms .............. 1 Report

[PLAT180_ALERT_4_G](javascript:makeHelpWindow(%22PLAT180.html%22)) Check Cell Rounding: # of Values Ending with 0 = 3 Note

[PLAT194_ALERT_1_G](javascript:makeHelpWindow(%22PLAT194.html%22)) Missing _cell_measurement_reflns_used Datum .... Please Add

[PLAT195_ALERT_1_G](javascript:makeHelpWindow(%22PLAT195.html%22)) Missing _cell_measurement_theta_max Datum .... Please Add

[PLAT196_ALERT_1_G](javascript:makeHelpWindow(%22PLAT196.html%22)) Missing _cell_measurement_theta_min Datum .... Please Add

[PLAT432_ALERT_2_G](javascript:makeHelpWindow(%22PLAT432.html%22)) Short Inter X...Y Contact O4 .. C3 .. 3.00 Ang.

[PLAT910_ALERT_3_G](javascript:makeHelpWindow(%22PLAT910.html%22)) Missing # of FCF Reflection(s) Below Theta(Min) 4 Note

[PLAT912_ALERT_4_G](javascript:makeHelpWindow(%22PLAT912.html%22)) Missing # of FCF Reflections Above STh/L= 0.600 9 Note

0 **ALERT level A** = Most likely a serious problem - resolve or explain

0 **ALERT level B** = A potentially serious problem, consider carefully

4 **ALERT level C** = Check. Ensure it is not caused by an omission or oversight

8 **ALERT level G** = General information/check it is not something unexpected
